# Investigating low birth weight and preterm birth as potential mediators in the relationship between prenatal infections and early child development: a linked administrative health data analysis

**DOI:** 10.1136/jech-2023-221826

**Published:** 2024-06-04

**Authors:** Iain Hardie, Aja Murray, Josiah King, Hildigunnar Anna Hall, Kenneth Okelo, Emily Luedecke, Louise Marryat, Lucy Thompson, Helen Minnis, Michael Lombardo, Philip Wilson, Bonnie Auyeung

**Affiliations:** 1 Department of Psychology, School of Philosophy Psychology and Language Sciences, The University of Edinburgh, Edinburgh, UK; 2 Centre for Health Security and Communicable Disease Control, Directorate of Health, Reykjavik, Iceland; 3 School of Health Sciences, University of Dundee, Dundee, UK; 4 Centre for Rural Health, University of Aberdeen, Inverness, UK; 5 Gillberg Neuropsychiatry Centre, University of Gothenburg, Gotherburg, Sweden; 6 School of Health and Wellbeing, University of Glasgow, Glasgow, UK; 7 Laboratory for Autism and Neurodevelopmental Disorders, Center for Neuroscience and Cognitive Systems, Istituto Italiano di Tecnologia, Rovereto, Italy; 8 Centre for Research and Education in General Practice, University of Copenhagen, Copenhagen, Denmark

**Keywords:** child health, infections, pregnancy

## Abstract

**Background:**

Prenatal infections are associated with childhood developmental outcomes such as reduced cognitive abilities, emotional problems and other developmental vulnerabilities. However, there is currently a lack of research examining whether this arises due to potential intermediary variables like low birth weight or preterm birth, or due to some other mechanisms of maternal immune activation arising from prenatal infections.

**Methods:**

Administrative data from the National Health Service health board of Greater Glasgow & Clyde, Scotland, were used, linking birth records to hospital records and universal child health review records for 55 534 children born from 2011 to 2015, and their mothers. Causal mediation analysis was conducted to examine the extent to which low birth weight and preterm birth mediate the relationship between hospital-diagnosed prenatal infections and having developmental concern(s) identified by a health visitor during 6–8 weeks or 27–30 months child health reviews.

**Results:**

Model estimates suggest that 5.18% (95% CI 3.77% to 7.65%) of the positive association observed between hospital-diagnosed prenatal infections and developmental concern(s) was mediated by low birth weight, while 7.37% (95% CI 5.36 to 10.88%) was mediated by preterm birth.

**Conclusion:**

Low birth weight and preterm birth appear to mediate the relationship between prenatal infections and childhood development, but only to a small extent. Maternal immune activation mechanisms unrelated to low birth weight and preterm birth remain the most likely explanation for associations observed between prenatal infections and child developmental outcomes, although other factors (for example, genetic factors) may also be involved.

**WHAT IS ALREADY KNOWN ON THIS TOPIC**

- Maternal infections during pregnancy are known to be associated with poorer childhood developmental outcomes, but it is not clear whether this association is driven by potential mediator variables like low birth weight or preterm birth.

**WHAT THIS STUDY ADDS**

- Low birth weight and preterm birth do appear to mediate the relationship between hospital-diagnosed maternal infections during pregnancy and poorer childhood developmental outcomes, but only to a small extent.

HOW THIS STUDY MIGHT AFFECT RESEARCH, PRACTICE OR POLICY

- Our study suggests that the relationship between maternal infections during pregnancy and poorer childhood developmental outcomes is likely to be driven mostly by mechanisms unrelated to low birthweight or preterm birth.
- Maternal immune activation (or mechanisms related to maternal immune activation) remains the most likely explanation for associations between maternal infections during pregnancy and developmental outcomes.
- Interventions aiming to improve childhood developmental outcomes should focus on mitigating the impact of MIA, rather than assuming that intervening on low birthweight and preterm birth will be sufficient

## Introduction

Maternal health during pregnancy plays an important role in shaping later childhood development, and there is now a growing body of evidence suggesting that prenatal maternal infections are associated with early childhood developmental outcomes. Previous research has found associations between maternal infections during pregnancy and reduced cognitive abilities, emotional difficulties, developmental concerns identified by health visitors and a range of other developmental vulnerabilities in children.^1–5^ There is also a large body of evidence linking prenatal maternal infections to childhood neurodevelopmental conditions, for example, autism and attention-deficit/hyperactivity disorder, as well as to adulthood mental health conditions including schizophrenia and bipolar disorder.^6–11^ It has been postulated that this arises from the immune response of mothers to prenatal infections, known as maternal immune activation (MIA), which creates a cascade of events affecting fetal brain development. This has been shown in animal models,^12 13^ and human studies are broadly consistent with this.^14^ Consequently, MIA tends to be cited as the most likely reason for associations observed between prenatal maternal infections and childhood developmental outcomes in existing research.^1–4^ MIA is not the only possible explanation for the association, as it is possible that both prenatal infections and childhood developmental issues may be caused by genetic factors. A lot of antenatal pyelonephritis (bacterial kidney infection), for example, is associated with congenital structural abnormalities in the renal tract, many of which are heritable, and there are well-known associations between minor dysmorphic features and developmental delays.^15 16^

**Figure 1:**
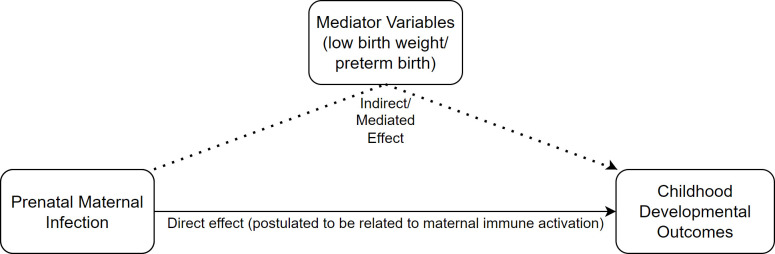
Diagram of the hypothesised relationship between prenatal infections, mediator variables and childhood developmental outcomes.

Nevertheless, if associations between prenatal infections and childhood development are caused by MIA rather than genetic factors, then MIA may exert its impact, in part, through some ‘mediator variables’ (ie, variables appearing in the causal sequence between two other variables^17^) like low birth weight or preterm birth. This is demonstrated in figure 1. Specifically, low birth weight and preterm birth may be mediator variables because some research suggests: (a) prenatal infections are associated with low birth weight and preterm birth^18–20^ and (b) preterm birth and low birth weight are in turn associated with poorer child developmental outcomes.^21–23^ Despite this, existing studies have tended to either ignore low birth weight and preterm birth from their modelling, or have included them in models as control variables, not mediators.^1–4 6^

Low birth weight and preterm birth are already considered factors that would flag risk of poorer developmental outcomes, and prenatal care already aims to prevent detrimental birth outcomes. However, by investigating how much of the relationship between prenatal infections and developmental outcomes is and is not mediated could still help to inform interventions in childhood development by improving our understanding of the mechanisms by which the relationship occurs. To examine this, the present study conducts a causal mediation analysis, using a large linked administrative health dataset from the National Health Service (NHS) health board of Greater Glasgow & Clyde, Scotland, to address the following research question:

To what extent do low birth weight and preterm birth mediate the relationship between hospital-diagnosed prenatal maternal infections and childhood developmental outcomes measured during child health reviews at age 6–8 weeks and age 27–30 months?

## Methods

### Data and participants

**Figure 2:**
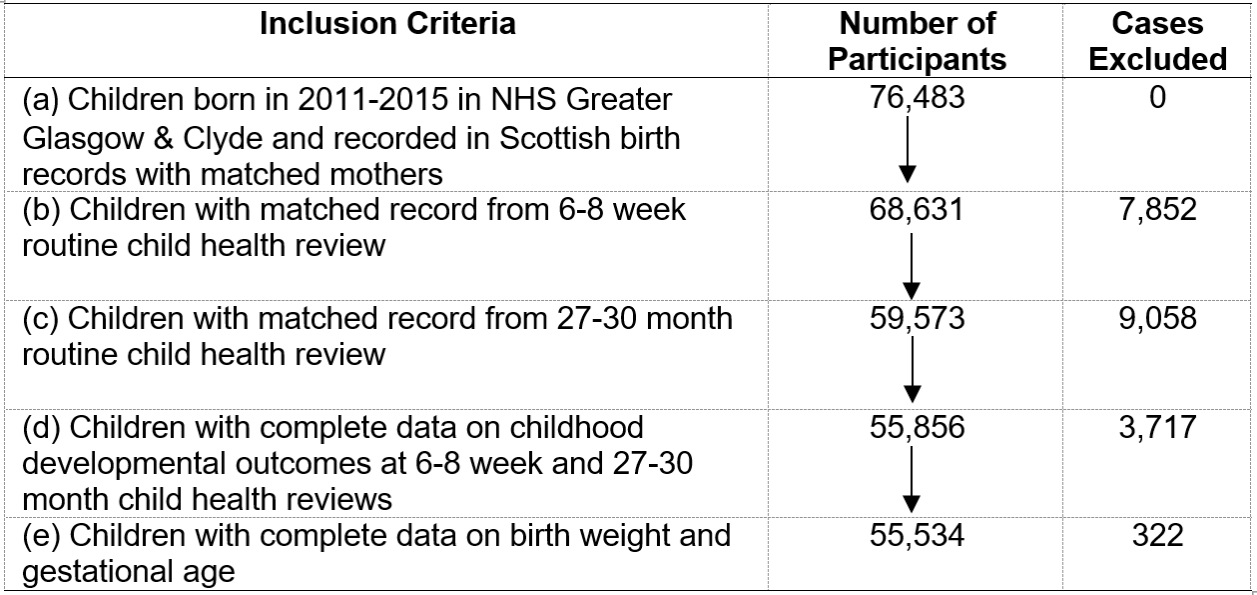
Flow chart of inclusion criteria, exclusions and number of participants.

The dataset comprised Scottish birth records, hospital records and routine child health review records. Participants were children born between 2011 and 2015 in NHS Greater Glasgow & Clyde, Scotland, along with their mothers. Full details of the inclusion criteria, and number of exclusions, are provided in figure 2. The final number of participants was 55 534 child-mother pairs.

### Measures

#### Outcome variables

In Scotland, all preschool children are eligible for routine health reviews, which are conducted by the NHS to review children’s health, development and well-being, and to provide advice and parenting support.^24 25^

Here, data from 6 to 8 weeks and 27 to 30 months health reviews were used to create a binary outcome variable indicating whether any (ie, at least one) childhood developmental concerns were identified by health visitors during these reviews (coded: 0=‘no’, 1=‘yes’). This could relate to developmental outcomes in any of the following domains: gross motor skills, hearing communication and vision social awareness (assessed at 6–8 week child health reviews), and personal-social, emotional-behavioural-attention and speech-language-communication (assessed at the 27–30 month child health reviews). Developmental concerns were defined as cases where health visitors categorised a developmental outcome as either ‘a concern’, ‘abnormal’ or ‘doubtful’ (ie, possible/likely abnormality). These judgements were made by health visitors through: (a) eliciting parental concerns, (b) making structured observations of the child and/or (c) using validated developmental questionnaires.^24^

Additional sensitivity analysis (outlined below) also included an additional outcome measure, ‘number of developmental concerns’. This was a numeric variable indicating the total number (from 0 to 6) of developmental concerns identified by health visitors.

#### Explanatory variable

The main explanatory variable was a binary measure of ‘hospital-diagnosed prenatal infections’ (coded: 0=‘no’, 1=‘yes’). Here, general and maternity hospital admission records^26 27^ were used to identify cases where mothers were diagnosed with at least one infection during pregnancy. This was done using ‘International Classification of Diseases 10’ (ICD10) infection codes. A full list of ICD10 codes included is provided in table 1. A list of the 20 most prevalent specific (ie, four digit) codes is also provided in online supplemental appendix 1.

10.1136/jech-2023-221826.supp1 Supplementary data

**Table 1:** List of ICD10 codes included in ‘hospital-diagnosed maternal infections during pregnancy’ variable

ICD10 codes	Type of infections included
A/B	Virus infections; bacterial infections; sexually transmitted infections/diseases
G0/G531/G630/G940	Meningitis; cranial nerve infections; polyneuropathy infections; hydrocephalus infections
H0/H1/H20/H22/H320/H440/H441/H451/H481	Eye infections
I30/I310/I311/I32/I33/I38/I39/I40/I41/I430/I520/ I521/I681/I980/I981	Pericarditis; endocarditis; myocarditis; heart infections; artery infections; cardiovascular infections
J0/J1/J2/J31/J32/J35/J36/J37/ J39/J40/J41/J42/J440/J85/J86	Sinusitis; tonsillitis; laryngitis; common cold; influenza; pneumonia; respiratory infections; bronchitis; rhinitis; pharyngitis; peritonsillar abscess; pharynx infections; pulmonary disease with infection; gangrene/lung abscess; pyothorax
K04/K05/K102/K112/K113/K122/K140/K35/K61/ K630/K65/K67/K75/K770/K81/K930	Periodontitis; pulpitis; gingivitis; necrosis; jaw inflammation/infection; salivary gland infection; cellulitis/mouth abscess; glossitis; appendicitis; anal infection/abscess; intestine abscess/infection; peritonitis; liver infection; cholecystitis; tuberculous of intestine
L0	Skin infections
M0	Septic arthritis
N080/N10/N11/N12/N22/N290/N291/N30/N330/ N34/N390/N61/N7	Glomerular disorders involving infection; interstitial nephritis; urinary calculus infection; kidney infection; cystitis; urethritis; urinary tract infection unspecified; infection in breast; pelvic/vaginal/vulva infection or inflammation
O23/O98	Infections/infectious disease associated with pregnancy
R50	Fever
T880	Infection following immunisation

ICD10, International Classification of Diseases 10.

Additional sensitivity analysis (outlined below) also included an alternative measure of the ‘hospital-diagnosed prenatal infections’, in which infections occurring in the month of childbirth were excluded.

#### Mediator variables

‘Low birth weight’ and ‘preterm birth’ were used as mediator variables. Low birth weight was defined as a binary variable using the WHO definition^28^ of weighing <2500 g (coded: 0=‘not low birth weight’, 1=‘low birth weight’). Similarly, preterm birth was a binary variable defined using the WHO definition^29^ of being born before 37 weeks of gestational age (coded: 0=‘not preterm’, 1=‘preterm’). Data on both variables were taken from Scottish birth records and maternity hospital records.

#### Control variables

Our analysis also included five control variables, which were made up of variables that were either: (a) confounders (ie, variables that are potentially associated with both likelihood of prenatal infections and childhood developmental outcomes, including proxies) or (b) key covariates (ie, important variables that are known to heavily influence childhood developmental outcomes). These were: (a) ‘sex of child’, a binary variable from birth records (coded: 0=‘male’, 1=‘female’), (b) ‘area-based deprivation’, a categorical variable indicating which Scottish Index of Multiple Deprivation (SIMD) quintile the child’s home postcode was in (coded: 1=most deprived, 2=more deprived, 3=medium deprived, 4=less deprived, 5=least deprived), (c) ‘maternal age’, a continuous variable, from maternity records, indicating the age of the child’s mother at time of childbirth, (d) ‘maternal prenatal smoking’, a binary variable, also from maternity records, indicating whether the child’s mother smoked during their pregnancy (coded: 0=‘no’, 1=‘yes’) and (e) ‘maternal history of mental health hospital admission’, a binary variable, from mental health hospital records, indicating whether the child’s mother had any record of historic mental health hospital admissions, that is, including all historic records up to 12 months after the birth of their child (coded: 0=‘no’, 1=‘yes’).

### Analysis

First, descriptive analysis was conducted in order to ascertain total frequencies of all variables included in the analysis, as well as frequencies across childhood development, prenatal infections and mediator variables.

Next, the mediation analysis was conducted to formally investigate whether the relationship between prenatal infections and childhood developmental concern(s) was mediated by the low birth weight or preterm birth variables. Causal mediation analysis, as a method, plays an important role in helping to determine how and why particular treatment effects arise by helping researchers to identify intermediate variables that occur in the pathway between a given treatment and a given outcome.^30^ Unlike conventional mediation analysis, causal mediation analysis attempts to better infer causality by focussing on non-parametric definitions of causal mediation effects, and is better able to achieve confounder control than conventional mediation analysis.^31^ It can also accommodate both linear and non-linear models and both continuous and discrete mediator/outcome variables.^31^ The analysis for the present study was specifically carried out using a ‘potential outcomes’ framework via the Stata/MP 16 ‘*mediation*’ package, which estimates mediation effects using Monte Carlo simulations.^30 32 33^ This works by simulating predicted values of a specified mediator or outcome variable and then calculating appropriate quantities of interest.^32^ Here, models were specified as follows using the ‘*mediation*’ package’s ‘medeff’ command^32 34 35^:

- Outcome: ‘developmental concern(s)’
- Treatment: ‘hospital-diagnosed prenatal infections’
- Mediators: ‘low birth weight’ and ‘preterm birth’ (in two separate models)
- Control variables: ‘sex of child’, ‘area-based deprivation’, ‘maternal age’, ‘maternal prenatal smoking’ and ‘maternal history of mental health hospital admission’

Based on the above, for the analysis of each mediator, the ‘medeff’ command runs two regression models (ie, a model for the mediator variable with prenatal infection as the treatment and sex of child’, ‘area-based deprivation’, ‘maternal age’, ‘maternal prenatal smoking’ as control variables, and a model for the developmental outcome variable with prenatal infection as the treatment and ‘sex of child’, ‘area-based deprivation’, ‘maternal age’, ‘maternal prenatal smoking’ and ‘maternal history of mental health hospital admission’ as control variables). From this, ‘medeff’ is able to provide summary estimates of the mediation, direct and total effects.^32^ As the above outcome and mediator variables were binary measures, logit models were used in our analysis. The same set of control variables, that is, all those listed above, were used to estimate predicted values for both the mediators and outcome. The only exception was that the maternal history of mental health hospital admissions control variable was omitted from the mediator model because it includes admissions from the first year of the child’s life and so temporally could have occurred after low birth weight/preterm birth had already occurred. Specifying the models like this gave estimates of the total effect of hospital-diagnosed prenatal infections on developmental concern(s), as well as the: (a) average direct effect (ADE), (b) average causal mediated effect (ACME) for each mediator variable and (c) percentage of the total effect that is mediated by each mediator variable.

In addition to the main analysis, two sensitivity analyses were conducted. First, the main analysis was repeated using the ‘number of developmental concern(s)’ outcome variable, as this potentially provides more detail into the extent of developmental concerns than the binary measure. As number of concern(s) is numeric, Ordinary Least Squares (OLS) modelling was used here for the outcome model rather than logit modelling. Second, the main analysis was repeated using the alternative measure of hospital-diagnosed prenatal infections which excluded infections from the month of childbirth. This was carried out because infections from the month of childbirth included, for example, group B *Streptococcus* carrier cases and herpes simplex virus encephalitis picked up when mothers were in hospital for delivery. These may be harmful for babies but are unlikely to cause MIA. Therefore, removing them may provide better insight into potential links between MIA and child development.

All analysis was conducted within Scotland’s National Safe Haven. Reporting is consistent with ‘REporting of studies Conducted using Observational Routinely collected health Data’ guidelines (for checklist see online supplemental appendix 2).^36^

## Results

### Descriptive statistics

Descriptive statistics are provided in online supplemental appendix 3. Overall, 21.2% of children had developmental concern(s). These were disproportionately prevalent among those exposed to hospital-diagnosed prenatal infection(s), those with low birth weight/born preterm and also among those who were male, from deprived areas and whose mother smoked during pregnancy or had mental health hospital admissions. Meanwhile, 5.1% of mothers had records of hospital-diagnosed infection(s) during pregnancy and these were more prevalent among those whose child had low birth weight or was born preterm, as well as among those from deprived areas, who smoked during pregnancy or had mental health hospital admissions. With regard to mediator variables, 7.0% of children in the dataset had low birth weight and 7.5% were born preterm. Finally, both preterm birth and low birth weight appeared to be more prevalent among those whose mothers smoked during pregnancy or had a history of mental health hospital admissions.

### Mediation analysis

Estimates from the mediation analysis are provided in table 2. The results highlight that there do appear to be mediating effects for both low birth weight and preterm birth, but only to a small extent. Specifically, the results highlight a positive association between hospital-diagnosed prenatal infections and developmental concern(s) (β=0.046 (95% CI 0.031 to 0.063) for total effect estimate). However, estimated mediation through low birth weight accounted for only 5.18% (95% CI 3.77% to 7.65%) of this (with an ACME estimate of β=0.002 (95% CI 0.001 to 0.004) compared with an ADE estimate of β=0.043 (95% CI 0.029 to 0.060)), while estimated mediation through preterm birth accounted for just an estimated 7.37% (95% CI 5.36% to 10.88%) of it (with an ACME estimate of β=0.003 (95% CI 0.002 to 0.005) compared with an ADE estimate of β=0.042 (95% CI 0.028 to 0.059)).

**Table 2:** Estimates of causal mediation in the relationship between hospital-diagnosed prenatal infection(s) and having one or more developmental concern(s) identified at age 6–8 weeks or age 27–30 months child health reviews

Mediator variable	Total effect (β)	Average direct effect (β)	Average causal mediated effect (β)	% of total effect mediated
Low birth weight	0.046 (0.031 to 0.063)	0.043 (0.029 to 0.060)	0.002 (0.001 to 0.003)	5.18% (3.77% to 7.65%)
Preterm birth	0.046 (0.031 to 0.0.063)	0.042 (0.028 to 0.059)	0.003 (0.002 to 0.004)	7.37% (5.36% to 10.88%)

β refers to beta-coefficient. 95% CIs are shown in parentheses underneath estimates. Models adjust for sex of child, area-based deprivation, maternal age, maternal prenatal smoking and maternal history of mental health hospital admission(s).

### Sensitivity analysis

Results of the sensitivity analysis, whereby the main analysis was repeated using the ‘number of developmental concern(s) variable as an additional outcome, are provided in online supplemental appendix 4. The results are broadly consistent with the main analysis.

Results of the sensitivity analysis in which the main analysis was repeated, but excluding hospital-diagnosed infections from the month of childbirth, are provided in online supplemental appendix 5. Here, the estimated total effect of hospital-diagnosed prenatal infections on developmental concern(s) was higher, possibly reflecting the fact that MIA was more likely to have occurred when using this measure (as many infections from the month of childbirth would be infections like group B *Streptococcus* carrier cases and herpes simplex virus encephalitis picked up when mothers are in hospital for delivery—these would not involve any MIA impacting fetal brain development). Estimated mediation effects were broadly similar to the main analysis.

## Discussion

This causal mediation analysis study used linked administrative health data for NHS Greater Glasgow & Clyde, Scotland, to investigate whether low birth weight and preterm birth mediate the relationship between hospital-diagnosed prenatal infections and early childhood developmental concern(s). The results highlight that low birth weight and preterm birth do appear to have mediating effects, but only to a small extent, mediating an estimated 5.18% (95% CI 3.77 to 7.65%) and 7.37% (95% CI 5.36 to 10.88%), respectively, of the relationship. Prior to the current analysis, previous studies had linked prenatal infections to adverse childhood developmental outcomes,^1–5^ and posited that this most likely occurred due to the MIA arising from prenatal infections. The present findings support this view, while also adding to previous studies by suggesting that only a small proportion of the association between prenatal infections and child development may be related to the mediating roles of low birth weight/preterm birth. Therefore, the majority of the association between prenatal infections and child development appears to occur due to other mechanisms—most likely mechanisms related to MIA. This suggests that interventions aiming to improve developmental outcomes should focus on mitigating the impact of MIA, rather than assuming that intervening on low birth weight and preterm birth will be sufficient.

A key strength of this study’s analysis is that it made use of a large linked administrative health dataset. However, there are some limitations. First, although the analysis controlled for measured confounders and uses a causal mediation framework, specifying the temporal sequencing of relationships, it is still difficult to disentangle the role of mediators from unmeasured confounding such as the effects of genetic factors (specifically the ‘downstream’ effects of genetics given that we have an implied temporal sequence with genetics being in place before mediator). For example, inherited syndromes associated with abnormalities in the urinary tract like renal coloboma syndrome may make prenatal infections more likely *and* make developmental concerns more likely and this may be unrelated to MIA. Due to this, it is possible that our estimates may overestimate direct and indirect effects. Moreover, it is possible that the potential mechanisms causing MIA to be linked to low birth weight/preterm birth are the same mechanisms involved in causing poorer developmental outcomes. In particular, MIA may be associated with neurobiology (eg, in a way which alters gene expression), which has two effects—one more proximal (inducing low birth weight/preterm birth) and one more distant (poorer developmental outcomes). If this is the case then mediation might make it look like MIA is having an effect on the more proximal outcome, and then the second indirect effect on developmental outcomes when in reality both could be linked to the similar prenatal effect but at different times. Another limitation is this study’s analysis tested mediators independently of each other. Given that low birth weight and preterm birth are correlated with each other, this makes it difficult to disentangle their effects.

There are also some general limitations to using administrative health datasets like the one used in the present study’s analysis. For example, there can be nuances around how ICD10 codes are applied and interpreted. The present study used an extensive list of ICD10 infection codes (table 1), but cannot rule out that infections data may be affected by variations in how codes are applied over time or between different medical professionals or reviewers who are entering the codes. This could impact the accuracy of our variables in picking up infections. Moreover, administrative records exclude people who do not interact with health services.^37–39^ Individuals from deprived areas are known to have lower participation rates in child health review data.^40^ Given that the present study was a complete case analysis, this potentially makes the dataset less representative of all child-mother pairs in Greater Glasgow & Clyde. Finally, the analysis was limited to data on infections from hospitals only, thus excluding infections that were less severe or treated within the community. Therefore, our study should be interpreted in the context of severe infections during pregnancy only.

To conclude, this causal mediation analysis study suggests that low birth weight and preterm birth may mediate the relationship between prenatal infections and childhood development, but only to a small extent. It remains the case that aspects of MIA unrelated to low birth weight and preterm birth are the most likely explanation for associations observed between prenatal infections and childhood developmental outcomes, although other factors, such as genetic factors, may be involved.

## Data Availability

Data may be obtained from a third party and are not publicly available. The administrative health datasets used for this study are not publicly available, but can be accessed via successfully applying to the NHS Scotland Public Benefit and Privacy Panel for Health and Social Care (HSC-PBPP). The authors of the present study were supported in applying for approval from HSC-PBPP by the electronic Data Research and Innovation Service (eDRIS) team at Public Health Scotland. eDRIS also facilitated access to the data via Scotland’s National Safe Haven.
