# Supplementary material for: Investigating low birth weight and preterm birth as potential mediators in the relationship between prenatal infections and early child development: a linked administrative health data analysis
Source: J Epidemiol Community Health. 2024 Jun 4;78(9):585–90. doi: 10.1136/jech-2023-221826 (PMC11347972; doi:10.1136/jech-2023-221826)
Supplement: Supplementary data [file jech-2023-221826supp001.pdf]

Supplemental Appendix 1. List of top 20 most commonly recorded maternal infections during pregnancy

ICD10 Code	Full description of infection	Number of infections recorded
O234	Unspecified infection of urinary tract in pregnancy	463
B951	Strep group B, as the cause of diseases classified in other chapters	274
O239	Other and unspecified genitourinary tract infection in pregnancy	231
O235	Infections of genital tract in pregnancy	218
J22X	Unspecified acute lower respiratory infection	178
A491	Streptococcal and enterococcal infection, site unspecified	173
N390	Urinary tract infection, site unspecified	166
N771	Vaginitis, vulvitis & vulvovaginitis in infectious and parasitic diseases	88
B373	Candidiasis of vulva and vagina	85
O230	Infections of kidney in pregnancy	74
B962	E. Coli as the cause of diseases classified in other chapters	61
B349	Viral infection, unspecified	44
B379	Candiasis, unspecified	43
A099	Gastroentiritis and colitis of unspecified origin	39
R509	Fever, unspecified	36
B24X	Unspecified human immunodeficiency virus [HIV] disease	32
L022	Cutaneous abscess, furuncle and carbuncle of trunk	29
N12X	Tubulo-interstitial nephritis, not specified as acute of chronic	27
B956	Staph aureus as the cause of diseases classified to other chapters	24
B169	Acute hepatitis B without delta-age	23

Notes: these are the top 20 most commonly recorded ICD10 four digit codes for infections during pregnancy for mothers in the sample. ICD10 codes listed in both general hospital admission records and maternity hospital admission records are included. Each hospital admission record could include up to six ICD10 codes, i.e. one for main condition recorded and up to five for other conditions recorded. Main conditions and all other conditions that correspond to infections during pregnancy period count towards overall number of infections listed.

Supplemental appendix 2. The RECORD statement – checklist of items, extended from the STROBE statement, that should be reported in observational studies using routinely collected health data.

	Item No.	STROBE items	Location in manuscript where items are reported	RECORD items	Location in manuscript where items are reported
**Title and abstract**					
	1	(a) Indicate the study's design with a commonly used term in the title or the abstract (b) Provide in the abstract an informative and balanced summary of what was done and what was found	Title: "linked administrative health data analysis" (p1)<br><br>Abstract: "administrative health data" and "causal mediation analysis" (p1)	RECORD 1.1: The type of data used should be specified in the title or abstract. When possible, the name of the databases used should be included.<br><br>RECORD 1.2: If applicable, the geographic region and timeframe within which the study took place should be reported in the title or abstract.<br><br>RECORD 1.3: If linkage between databases was conducted for the study, this should be clearly stated in the title or abstract.	Title/abstract: "administrative health data" (p1)<br><br>Abstract: "Greater Glasgow & Clyde" and "children born 2011-2015" (p1)<br><br>Abstract: "linking birth records to hospital records for 55,534 children" (p1)
**Introduction**					
Background rationale	2	Explain the scientific background and rationale for the investigation being reported	Intro: "existing studies tend to either ignore low birthweight and preterm birth from their modelling or..." (p2)		
Objectives	3	State specific objectives, including any prespecified hypotheses	Intro: "research question: To what extent to low birthweight and preterm birth mediate the relationship..." (p2)		
**Methods**					
Study Design	4	Present key elements of study design early in the paper	Research design: "causal mediation analysis of..." (p3)		
Setting	5	Describe the setting, locations, and relevant dates, including periods of recruitment, exposure, follow-up, and data collection	Data and participants: "children born 2011-2015 in NHS Greater Glasgow & Clyde, Scotland, and their mothers" (p3)		

Participants	6	*(a) Cohort study* - Give the eligibility criteria, and the sources and methods of selection of participants. Describe methods of follow-up<br>*Case-control study* - Give the eligibility criteria, and the sources and methods of case ascertainment and control selection. Give the rationale for the choice of cases and controls<br>*Cross-sectional study* - Give the eligibility criteria, and the sources and methods of selection of participants<br><br>*(b) Cohort study* - For matched studies, give matching criteria and number of exposed and unexposed<br>*Case-control study* - For matched studies, give matching criteria and the number of controls per case	Figure 2 sets out inclusion criteria and number of participants in full (p3)	RECORD 6.1: The methods of study population selection (such as codes or algorithms used to identify subjects) should be listed in detail. If this is not possible, an explanation should be provided.<br><br>RECORD 6.2: Any validation studies of the codes or algorithms used to select the population should be referenced. If validation was conducted for this study and not published elsewhere, detailed methods and results should be provided.<br><br>RECORD 6.3: If the study involved linkage of databases, consider use of a flow diagram or other graphical display to demonstrate the data linkage process, including the number of individuals with linked data at each stage.	Done in Figure 2 (p3)
Variables	7	Clearly define all outcomes, exposures, predictors, potential confounders, and effect modifiers. Give diagnostic criteria, if applicable.	Done in measures section (p3-4)	RECORD 7.1: A complete list of codes and algorithms used to classify exposures, outcomes, confounders, and effect modifiers should be provided. If these cannot be reported, an explanation should be provided.	Done in measures section (p3-4)
Data sources/ measurement	8	For each variable of interest, give sources of data and details of methods of assessment (measurement).<br>Describe comparability of assessment methods if there is more than one group	Done in measures section (p3-4)		

Bias	9	Describe any efforts to address potential sources of bias	Control variables and sensitivity analysis used (set out p4-6)		
Study size	10	Explain how the study size was arrived at	Done in Figure 2 (p3)		
Quantitative variables	11	Explain how quantitative variables were handled in the analyses. If applicable, describe which groupings were chosen, and why	Done in measures section (p3-4)		
Statistical methods	12	(a) Describe all statistical methods, including those used to control for confounding<br>(b) Describe any methods used to examine subgroups and interactions<br>(c) Explain how missing data were addressed<br>(d) *Cohort study* - If applicable, explain how loss to follow-up was addressed<br>*Case-control study* - If applicable, explain how matching of cases and controls was addressed<br>*Cross-sectional study* - If applicable, describe analytical methods taking account of sampling strategy<br>(e) Describe any sensitivity analyses	All done in analysis section (p4-6)		
Data access and cleaning methods		..		RECORD 12.1: Authors should describe the extent to which the investigators had access to the database population used to create the study population.	Done in methods section (p3-6)

				RECORD 12.2: Authors should provide information on the data cleaning methods used in the study.	Done in methods section (p3-6)
Linkage		..		RECORD 12.3: State whether the study included person-level, institutional-level, or other data linkage across two or more databases. The methods of linkage and methods of linkage quality evaluation should be provided.	Done in methods section and Figure 2. Also discussed in discussion section (p3-6 and p11-12)
**Results**					
Participants	13	(a) Report the numbers of individuals at each stage of the study (*e.g.*, numbers potentially eligible, examined for eligibility, confirmed eligible, included in the study, completing follow-up, and analysed)<br>(b) Give reasons for non-participation at each stage.<br>(c) Consider use of a flow diagram	Done in Figure 2 (p3)	RECORD 13.1: Describe in detail the selection of the persons included in the study (*i.e.,* study population selection) including filtering based on data quality, data availability and linkage. The selection of included persons can be described in the text and/or by means of the study flow diagram.	Done in Figure 2 and in text (p3)
Descriptive data	14	(a) Give characteristics of study participants (*e.g.*, demographic, clinical, social) and information on exposures and potential confounders<br>(b) Indicate the number of participants with missing data for each variable of interest<br>(c) *Cohort study* - summarise follow-up time (*e.g.*, average and total amount)	Done in Table 2 (p8-9)		
Outcome data	15	*Cohort study* - Report numbers of outcome events or summary measures over time<br>*Case-control study* - Report numbers in each exposure	Done in Table 2 (p8-9)		

		category, or summary measures of exposure<br>*Cross-sectional study* - Report numbers of outcome events or summary measures			
Main results	16	(a) Give unadjusted estimates and, if applicable, confounder-adjusted estimates and their precision (e.g., 95% confidence interval). Make clear which confounders were adjusted for and why they were included<br>(b) Report category boundaries when continuous variables were categorized<br>(c) If relevant, consider translating estimates of relative risk into absolute risk for a meaningful time period	Done in Table 3 and in text (p7 and p10)		
Other analyses	17	Report other analyses done—e.g., analyses of subgroups and interactions, and sensitivity analyses	Done in sensitivity analysis section (p7-11)		
**Discussion**					
Key results	18	Summarise key results with reference to study objectives	Done at start of discussion (p11)		
Limitations	19	Discuss limitations of the study, taking into account sources of potential bias or imprecision. Discuss both direction and magnitude of any potential bias	Done towards end of discussion (p11-12)	RECORD 19.1: Discuss the implications of using data that were not created or collected to answer the specific research question(s). Include discussion of misclassification bias, unmeasured confounding, missing data, and changing eligibility over time, as they pertain to the study being reported.	Done in limitations discussion (p11-12)
Interpretation	20	Give a cautious overall interpretation of results considering objectives,	Done in discussion (p11-12)		

		limitations, multiplicity of analyses, results from similar studies, and other relevant evidence			
Generalisability	21	Discuss the generalisability (external validity) of the study results	Done in discussion (p11-12)		
**Other Information**					
Funding	22	Give the source of funding and the role of the funders for the present study and, if applicable, for the original study on which the present article is based	Done in acknowledgements (p12)		
Accessibility of protocol, raw data, and programming code		..		RECORD 22.1: Authors should provide information on how to access any supplemental information such as the study protocol, raw data, or programming code.	Data cannot be shared as access requires approval from the NHS HSC-PBPP panel

*Reference: Benchimol EI, Smeeth L, Guttmann A, Harron K, Moher D, Petersen I, Sørensen HT, von Elm E, Langan SM, the RECORD Working Committee. The REporting of studies Conducted using Observational Routinely-collected health Data (RECORD) Statement. *PLoS Medicine* 2015; in press.

*Checklist is protected under Creative Commons Attribution (CC BY) license.

Supplemental appendix 3. Descriptive statistics of participants by childhood development, prenatal infections and mediator variables

		Childhood development		Prenatal infection(s)		Mediator variables			
		One or more childhood developmental concerns identified by health visitor		Hospital-diagnosed prenatal infection(s)		Low birthweight		Preterm birth	
	Total	*No*	*Yes*	*No*	*Yes*	*No*	*Yes*	*No*	*Yes*
**Childhood development**									
One or more childhood developmental concerns identified by health visitor									
No	43,771 (78.8%)			41,703 (79.1%)	2,842 (72.8%)	41,022 (79.4%)	2,749 (70.7%)	40,809 (79.4%)	2,962 (71.4%)
Yes	11,763 (21.2%)			10,989 (20.9%)	774 (27.2%)	10,625 (20.6%)	1,138 (29.3%)	10,574 (20.6%)	1,189 (28.6%)
**Prenatal infections**									
Hospital-diagnosed prenatal infection(s)									
No	52,692 (94.9%)	41,703 (95.3%)	10,989 (93.4%)			49,102 (95.1%)	3,590 (92.4%)	48,887 (95.1%)	3,805 (91.7%)
Yes	2,842 (5.1%)	2,068 (4.7%)	774 (6.6%)			2,545 (4.9%)	297 (7.6%)	2,496 (4.9%)	346 (8.3%)
**Mediator variables**									
Low birthweight									
No	51,647 (92.0%)	40,809 (93.2%)	10,625 (90.3%)	49,102 (93.2%)	2,545 (89.6%)			49,945 (97.2%)	1,702 (41.0%)
Yes	3,887 (7.0%)	2,962 (6.8%)	1,138 (9.7%)	3,590 (6.8%)	297 (10.4%)			1,438 (2.8%)	2,449 (59.0%)
Preterm birth									
No	51,383 (92.5%)	40,809 (93.2%)	10,574 (89.9%)	48,887 (92.8%)	2,496 (87.8%)	49,945 (96.7%)	1,438 (37.0%)		
Yes	4,151 (7.5%)	2,962 (6.8%)	1,189 (10.1%)	3,805 (7.2%)	346 (12.2%)	1,702 (3.3%)	2,449 (63.0%)		
**Confounder/covariates**									
Sex of child									
Male	28,186	20,518	7,668	26,722	1,464	26,392	1,794	25,972	2,214

	(50.7%)	(46.9%)	(65.2%)	(50.7%)	(51.5%)	(51.1%)	(46.2%)	(50.5%)	(53.3%)
Female	27,348 (49.3%)	23,253 (53.1%)	4,095 (34.8%)	25,970 (49.3%)	1,378 (48.5%)	25,255 (48.9%)	2,093 (53.8%)	25,411 (49.5%)	1,937 (46.7%)
Area-based deprivation									
1 (most deprived)	22,117 (39.8%)	16,353 (37.4%)	5,764 (49.0%)	20,787 (39.4%)	1,330 (46.8%)	20,469 (39.6%)	1,648 (42.4%)	20,489 (39.9%)	1,628 (39.2%)
2 (more deprived)	10,261 (18.5%)	7,981 (18.2%)	2,280 (19.4%)	9,713 (18.4%)	548 (19.3%)	9,492 (18.4%)	769 (19.8%)	9,434 (18.4%)	827 (19.9%)
3 (medium deprived)	8,405 (15.1%)	6,804 (15.5%)	1,601 (13.6%)	8,006 (15.2%)	399 (14.0%)	7,834 (15.2%)	571 (14.7%)	7,751 (15.1%)	654 (15.8%)
4 (less deprived)	7,126 (12.8%)	5,988 (13.7%)	1,138 (9.7%)	6,785 (12.9%)	341 (12.0%)	6,709 (13.0%)	417 (10.7%)	6,642 (12.9%)	484 (11.6%)
5 (least deprived)	7,625 (13.7%)	6,645 (15.2%)	980 (8.3%)	7,401 (14.5%)	224 (7.9%)	7,143 (13.8%)	482 (12.4%)	7,067 (13.8%)	558 (13.4%)
Maternal age									
Mean age	29.6 years	29.9 years	28.5 years	29.7 years	28.4 years	29.6 years	29.9 years	29.5 years	30.1 years
Maternal prenatal smoking									
No	47,647 (85.5%)	38,393 (87.7%)	9,254 (78.7%)	45,376 (86.1%)	2,271 (79.9%)	44,767 (86.7%)	2,880 (74.1%)	44,257 (86.1%)	3,390 (81.7%)
Yes	7,887 (14.2%)	5,378 (12.3%)	2,509 (21.3%)	7,316 (13.9%)	571 (20.1%)	6,880 (13.3%)	1,007 (25.9%)	7,126 (13.9%)	761 (18.3%)
Maternal history of mental health hospital admissions									
No	54,652 (98.4%)	43,173 (98.6%)	11,479 (97.6%)	51,888 (98.5%)	2,764 (97.3%)	50,879 (98.5%)	3,773 (97.1%)	50,609 (98.5%)	4,043 (97.4%)
Yes	882 (1.6%)	598 (1.37%)	284 (2.4%)	804 (1.5%)	78 (2.7%)	768 (1.5%)	114 (2.9%)	774 (1.5%)	108 (2.6%)

*Notes: Table shows frequencies and percentages for categorical or binary variables, and mean values for continuous variable.

Supplemental Appendix 4. Sensitivity analysis estimates of causal mediation in the relationship between hospital-diagnosed prenatal infection(s) and number of developmental concern(s) identified at age 6-8 weeks or age 27-30 months child health reviews

Mediator Variable	Total Effect (β)	Average Direct Effect (β)	Average Causal Mediated Effect (β)	% of Total Effect Mediated
Low Birthweight	0.101 [0.075-0.129]	0.096 [0.071-0.123]	0.005 [0.003-0.007]	4.59% [3.59%-6.15%]
Preterm Birth	0.101 [0.075-0.129]	0.094 [0.069-0.121]	0.007 [0.005-0.009]	6.77% [5.30%-9.09%]

*Notes: β refers to beta coefficient. 95% confidence intervals are shown in square brackets underneath estimates. Models adjust for sex of child, area-based deprivation, maternal age, maternal prenatal smoking and maternal history of mental health hospital admission(s).

BMJ Publishing Group Limited (BMJ) disclaims all liability and responsibility arising from any reliance placed on this supplemental material which has been supplied by the author(s)

Supplemental Appendix 5. Sensitivity analysis estimates of causal mediation in the relationship between hospital-diagnosed prenatal infection(s) and having one or more developmental concern(s) identified at age 6-8 week or age 27-30 month child health reviews (infections in month of childbirth excluded)

Mediator Variable	Total Effect (Q)	Average Direct Effect (Q)	Average Causal Mediated Effect (Q)	% of Total Effect Mediated
Low Birthweight	0.057 [0.037-0.081]	0.055 [0.035-0.078]	0.003 [0.001-0.004]	5.48% [3.48%-7.76%]
Preterm Birth	0.057 [0.037-0.081]	0.054 [0.034-0.077]	0.003 [0.002-0.005]	5.85% [4.16%-9.01%]

*Notes: β refers to beta coefficient. 95% confidence intervals are shown in square brackets underneath estimates. Models adjust for sex of child, area-based deprivation, maternal age, maternal prenatal smoking and maternal history of mental health hospital admission(s).
